# Improving the Generalizability of Infantile Cataracts Detection via Deep Learning-Based Lens Partition Strategy and Multicenter Datasets

**DOI:** 10.3389/fmed.2021.664023

**Published:** 2021-05-07

**Authors:** Jiewei Jiang, Shutao Lei, Mingmin Zhu, Ruiyang Li, Jiayun Yue, Jingjing Chen, Zhongwen Li, Jiamin Gong, Duoru Lin, Xiaohang Wu, Zhuoling Lin, Haotian Lin

**Affiliations:** ^1^School of Electronic Engineering, Xi'an University of Posts and Telecommunications, Xi'an, China; ^2^School of Communications and Information Engineering, Xi'an University of Posts and Telecommunications, Xi'an, China; ^3^School of Mathematics and Statistics, Xidian University, Xi'an, China; ^4^State Key Laboratory of Ophthalmology, Zhongshan Ophthalmic Center, Sun Yat-sen University, Guangzhou, China

**Keywords:** lens partition strategy, infantile cataracts, automatic diagnosis, Faster R-CNN, multicenter slit-lamp images

## Abstract

Infantile cataract is the main cause of infant blindness worldwide. Although previous studies developed artificial intelligence (AI) diagnostic systems for detecting infantile cataracts in a single center, its generalizability is not ideal because of the complicated noises and heterogeneity of multicenter slit-lamp images, which impedes the application of these AI systems in real-world clinics. In this study, we developed two lens partition strategies (LPSs) based on deep learning Faster R-CNN and Hough transform for improving the generalizability of infantile cataracts detection. A total of 1,643 multicenter slit-lamp images collected from five ophthalmic clinics were used to evaluate the performance of LPSs. The generalizability of Faster R-CNN for screening and grading was explored by sequentially adding multicenter images to the training dataset. For the normal and abnormal lenses partition, the Faster R-CNN achieved the average intersection over union of 0.9419 and 0.9107, respectively, and their average precisions are both > 95%. Compared with the Hough transform, the accuracy, specificity, and sensitivity of Faster R-CNN for opacity area grading were improved by 5.31, 8.09, and 3.29%, respectively. Similar improvements were presented on the other grading of opacity density and location. The minimal training sample size required by Faster R-CNN is determined on multicenter slit-lamp images. Furthermore, the Faster R-CNN achieved real-time lens partition with only 0.25 s for a single image, whereas the Hough transform needs 34.46 s. Finally, using Grad-Cam and t-SNE techniques, the most relevant lesion regions were highlighted in heatmaps, and the high-level features were discriminated. This study provides an effective LPS for improving the generalizability of infantile cataracts detection. This system has the potential to be applied to multicenter slit-lamp images.

## Introduction

Artificial intelligence (AI) algorithms, especially deep learning, hold great promise in the automatic diagnosis of extensive diseases based on medical images, such as infantile cataracts (1–4), diabetic retinopathy (5, 6), age-related macular degeneration (7, 8), glaucoma (9), breast cancer (10, 11), skin cancer (12), and autism spectrum disorder (13). These applications indicate that deep learning has sufficient capabilities to provide high-quality healthcare services, solve time-consuming and labor-intensive problems in manual diagnosis, and alleviate the uneven distribution of medical resources.

The previous studies mainly focused on the comparison and selection of different classifiers, directly performing automatic diagnoses based on the original images (5–13), or applying a simple preprocessing method to obtain the approximate contour of the lesion region (1–4). However, the precision of the lesion region partition directly affects the performance of the diagnosis model, especially for a lesion region surrounded by lots of noises. The quantitative evaluation of the impact of automatic partition strategies on diagnosis models was not well-investigated. In addition, although transfer learning and data augmentation techniques were applied to train ultradeep convolutional neural networks (CNNs) based on medical images (14–17), the number of available medical images is far less than that of natural images, especially for rare diseases (18–21). And the complicated noises and heterogeneity of multicenter images affect the diagnosis result. Based on limited medical images, the generalizability of deep learning on multicenter medical images is still unclear. Therefore, it is urgent to compare and analyze the impacts of different partition strategies and multicenter images on the automatic diagnosis system.

Infantile cataract is a common and serious ophthalmic disease for infants and young children, which often causes irreversible visual impairment or blindness if it is not diagnosed and treated in time (22–25). Ophthalmological diagnosis is a time-consuming, labor-intensive, and subjective process. To more accurately identify the severities of infantile cataracts using AI methods, our team designed a new grading method for infantile cataracts in the previous research (1). The severities of infantile cataracts were graded from three perspectives, including opacity area (extensive vs. limited), density (dense vs. transparent), and location (central vs. peripheral). Lens opacity area that covers more than 50% of the pupil is defined as extensive; otherwise, it is defined as limited. Lens opacity density that completely blocks the light is labeled as dense; otherwise, it is defined as transparent. Lens opacity location that fully covers the visual axis of the pupil is called central; otherwise, it is called peripheral. For the area, density, and location of the lens opacity, this grading method does not need to consider the specific types of infantile cataracts, but can cover almost all types of infantile cataracts. And it is more suitable for the processing flow of AI algorithms.

Based on this grading method, we have developed an automatic diagnosis system CC-Cruiser (1, 3) based on CNNs, which achieved satisfactory accuracy for infantile cataracts detection based on a single-center dataset derived from Zhongshan Ophthalmic Center (ZOC) of Sun Yat-Sen University. The Canny detection and Hough transform (26, 27) were used to localize the lens region and eliminate noises such as iris, eyelids, eyelashes, etc. However, the partitioned lens region was not completely consistent with the true lens region, and the impact of the partition strategy on the CC-Cruiser was not studied. Recently, our team applied and extended the CC-Cruiser to other four multicenter slit-lamp images (28). However, the comparative experiments demonstrated that the performance of the CC-Cruiser in multicenter was inferior to that in single-center, and it was even weaker than that of senior ophthalmologists based on a multicenter randomized controlled trial. These studies indicated that although CC-Cruiser achieved satisfactory accuracy for infantile cataracts detection in single-center testing, its generalizability was weak on the multicenter datasets. Therefore, the slit-lamp images of infantile cataracts provide an ideal application scenario for us to study the impact of partition strategies and multicenter datasets on the generalizability of the diagnosis system.

In this study, we first collected slit-lamp images from other four independent ophthalmic clinics to construct multicenter datasets. Then, we proposed lens partition strategies (LPSs) based on deep learning Faster R-CNN and Hough transform for localizing the lens region and explored the impacts of different LPSs on the performance of the diagnosis system. Furthermore, we divided the partitioned multicenter dataset into five equal size subsets and sequentially added one to four subsets into the CC-Cruiser training dataset to analyze the impact of the sample size of multicenter slit-lamp images on the generalizability of the diagnosis system. Finally, we obtained a clinically applicable LPS and minimal training sample size for infantile cataracts based on multicenter slit-lamp images.

## Materials and Methods

### Datasets and Participants

As shown in Table 1, the slit-lamp dataset consists of two parts. The first part used to train CC-Cruiser was derived between April 2013 and February 2015 from ZOC of Sun Yat-Sen University, including 476 normal images and 410 infantile cataract images (1, 3). The second part included 336 normal images and 421 infantile cataract images that were obtained between August 2017 and May 2018 from the other four clinical institutions (the Central Hospital of Wuhan, Shenzhen Eye Hospital, Kaifeng Eye Hospital, and the Second Affiliated Hospital of Fujian Medical University) distributed in different regions across China (28). Furthermore, the baseline demographics and clinical features of the two datasets are summarized in Table 1. Each slit-lamp image was comprehensively evaluated and labeled by three senior ophthalmologists from three-degree grading: opacity area (extensive vs. limited), density (dense vs. transparent), and location (central vs. peripheral). The definition of the three-degree grading was consistent with previous studies (1, 3, 28, 29).

**Table 1:** Baseline demographics and clinical features of slit-lamp datasets.

**Item**	**CC-Cruiser dataset**	**Multicenter dataset**
No. of subjects	536	433
Age (years)^*^	4.25 (0.36)	6.24 (0.45)
No. (%) of male	299 (55.8%)	196 (45.3%)
No. (%) of family history of cataracts	46 (8.6%)	28 (6.5%)
Total no. of images	886	757
No. (%) of images with cataracts	410 (46.3%)	421 (55.6%)
Opacity area		
Extensive^#^	238 (58.0%)	261 (62.0%)
Limited^#^	172 (42.0%)	160 (38.0%)
Opacity density		
Dense^#^	231 (56.3%)	283 (67.2%)
Transparent^#^	179 (43.7%)	138 (32.8%)
Opacity location		
Central^#^	260 (63.4%)	254 (60.3%)
Peripheral^#^	150 (36.6%)	167 (39.7%)

* *Data are presented as the mean (standard deviation)*.

# *data are no. (%) of images*.

Recruited participants were required to meet the standard inclusion criteria of the above five ophthalmic clinics. Participants were eligible for this study when they were younger than 14 years, with or without eye symptoms, and had no history of any eye surgeries. All participants were required to undergo a slit-lamp imaging examination. If necessary, the sedative was given to small, uncooperative infants and young children. All slit-lamp images containing a clear lens were enrolled in this study. Low-quality images were excluded from this study.

Written informed consent was obtained from at least one guardian of each participant according to the Childhood Cataract Program of the Chinese Ministry of Health (18) when the slit-lamp image was enrolled. In the study, all personal privacy information of participants was removed, and only the slit-lamp images were retained. The research protocol was approved by the institutional review board/ethics committee of ZOC.

### Study Design and Procedures

This research mainly focused on two aspects: LPS and multicenter slit-lamp images. Specifically, two automatic LPSs were proposed and compared to obtain an optimal partition strategy. Then, multicenter slit-lamp images were divided into five equal size subsets, which were sequentially added to the single-center training dataset of CC-Cruiser to investigate the impact of the sample size of multicenter datasets on the generalizability of the diagnosis models.

**Figure 1:**
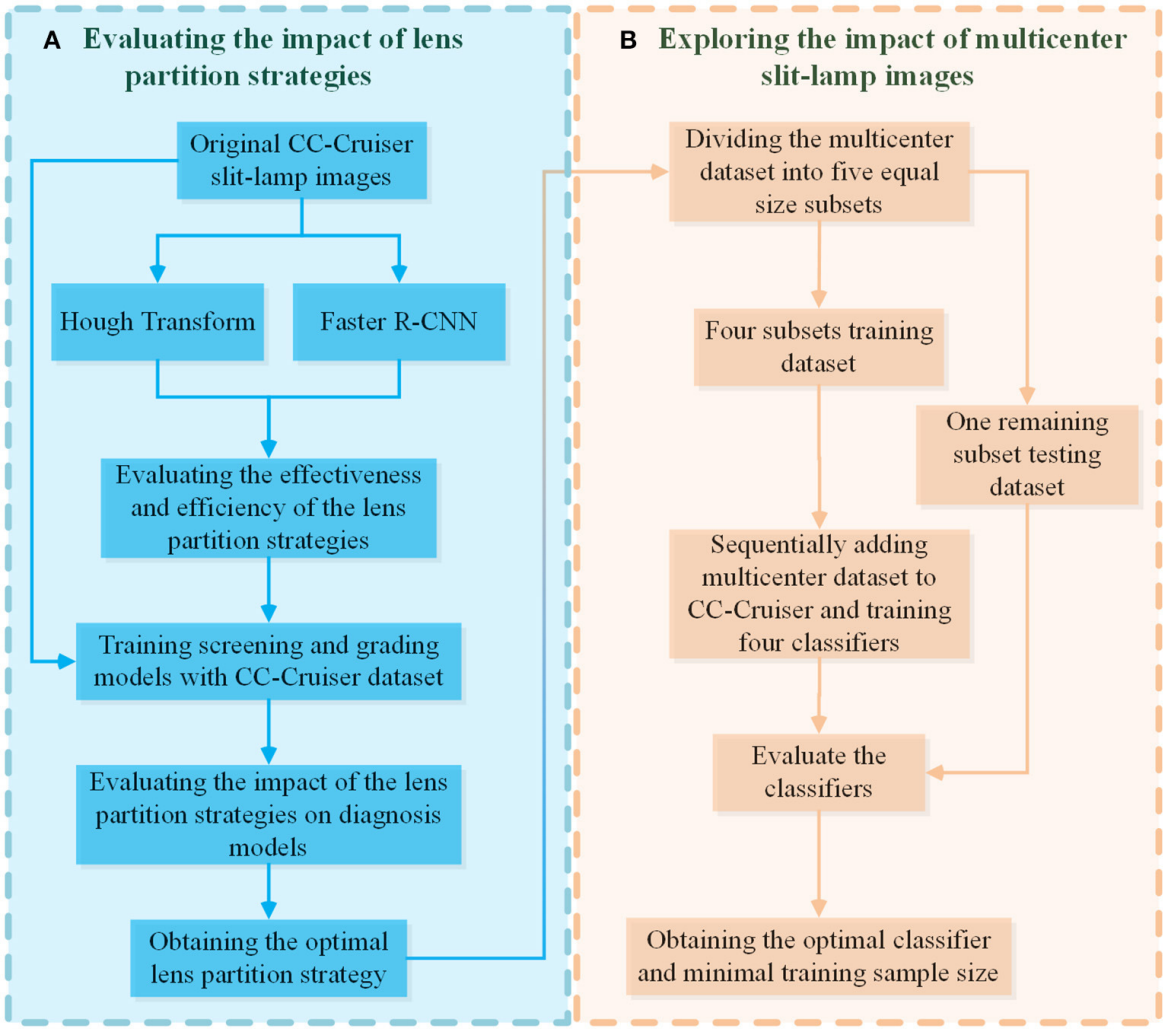
The evaluation pipeline for the impacts of LPSs and multicenter slit-lamp images. **(A)** The evaluation module of the impact of the LPSs. **(B)** The evaluation module of the impact of multicenter slit-lamp images. LPSs, lens partition strategies.

As shown on the left side of Figure 1, two LPSs based on deep learning Faster R-CNN and Hough transform were implemented to localize the normal and abnormal lenses in the CC-Cruiser dataset, and their performance was compared in terms of partition accuracy and efficiency. Then, based on the original dataset and two types of partitioned datasets, the models for infantile cataracts screening and three-degree grading were trained and evaluated using 5-fold cross-validation (30). After obtaining the optimal lens partition, as shown on the right side of Figure 1, the multicenter slit-lamp images were divided into five equal size subsets. One subset was used for testing, and the other four subsets were added to the CC-Cruiser training dataset in a piece-by-piece manner. Thus, including the original model without adding any multicenter training dataset, a total of five classifiers were trained. Finally, the performance of five classifiers was compared to evaluate the impact of the multicenter slit-lamp images on the diagnosis model. The optimal classifier and appropriate sample size were obtained.

### Overall Architecture of LPS and Infantile Cataracts Diagnosis

**Figure 2:**
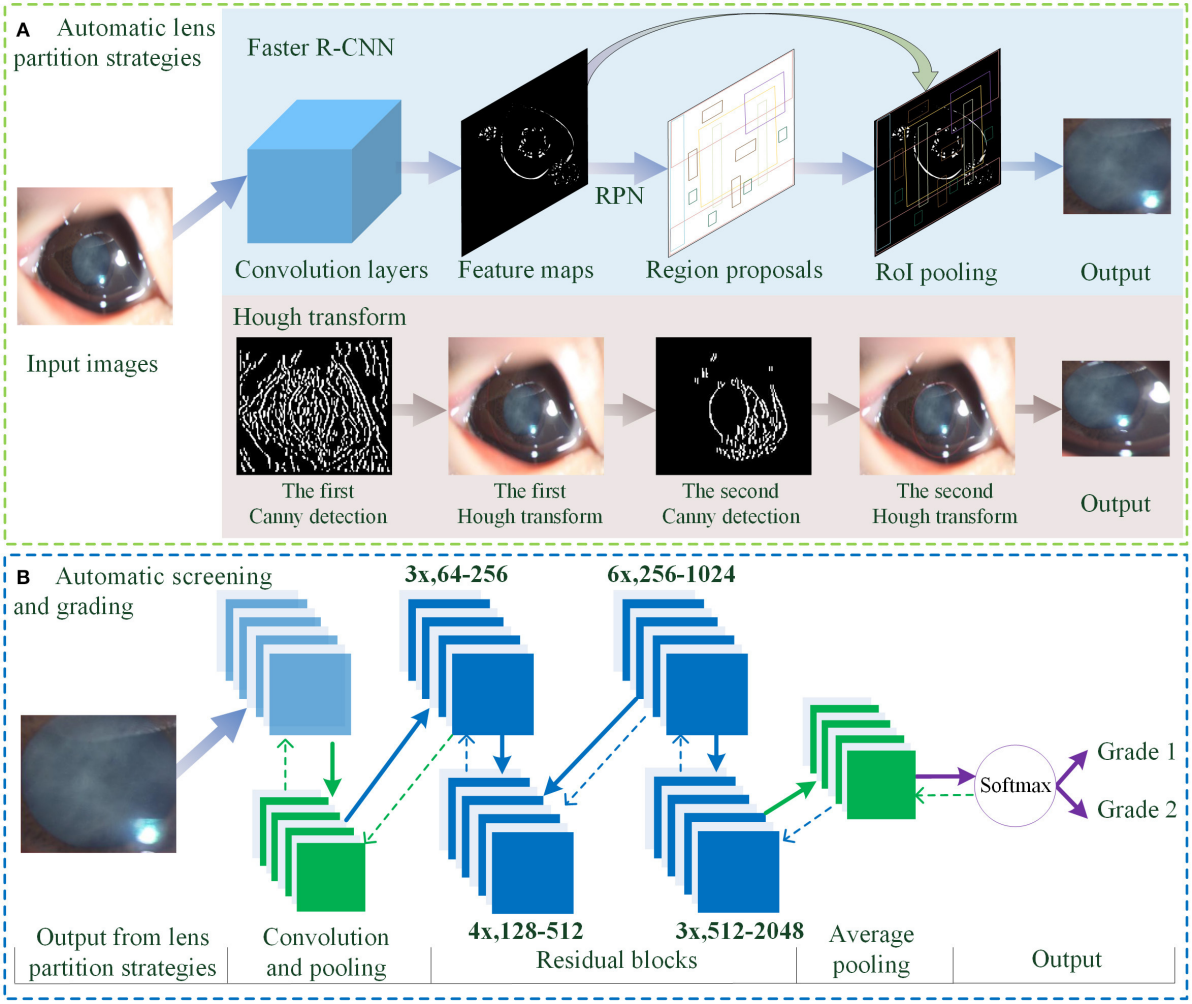
The overall architecture of LPS and diagnosis for infantile cataracts. **(A)** The automatic LPSs using Faster R-CNN and Hough transform. **(B)** The automatic screening and grading of infantile cataracts using the ResNet50. LPS, lens partition strategy; RPN, region proposal network; RoI, region of interest.

As shown in Figure 2, the overall architecture consists of two modules: automatic LPS and infantile cataracts screening and grading. Two LPSs are present to localize the lens regions on the original slit-lamp images (Figure 2A). Then, the partitioned lens regions are input into a 50-layer residual CNN (ResNet5) for screening and three-degree grading of infantile cataracts (Figure 2B).

For lens partition, consistent with our previous studies (1, 3, 4), the twice-applied Canny detection and Hough transform are first used to detect the lens region. As shown in Figure 2A, the general contour of the lens is identified on the H component of HSV (hue, saturation, and value) using the first round of the Hough transform, and then the finer boundary of the lens is partitioned on the S component of HSV using the second round of the Hough transform. In this study, an alternative partition strategy based on the Faster R-CNN method (31) is proposed for detecting lens regions (Figure 2A). The feature maps show the output of the convolution layers. The region proposal network employs an anchor mechanism to generate a batch of region proposals of the normal and abnormal lens on the feature maps. The region of interest (RoI) pooling performs maximum pooling on the region proposals with non-uniform sizes to produce the fixed-size feature maps. Each region proposal is compared with the true lens region marked by ophthalmologists. Two stages of the Faster R-CNN are applied to predict the boundary coordinates of the lens region. As the backbone network, a pretrained 50-layer ResNet model (32) based on the ImageNet dataset was used in the Faster R-CNN for lens partition.

In the automatic diagnosis of infantile cataracts (1, 3, 4) and multiple ophthalmic disorders (33–35), the AlexNet (36), GoogleNet (37), and ResNet (32) were performed and compared in detail, and the superiority of the ResNet was verified. In this study, a 50-layer ResNet classifier is applied for infantile cataracts screening and three-degree grading (Figure 2B). The overall architecture of the ResNet mainly consists of convolution layers, max-pooling operation, 16 residual blocks, batch normalization technique, average pooling, softmax layer, transfer learning, and data augmentation technique. The residual blocks are used to address the degradation problem caused by the ultra-deep networks (32, 38). As shown in Figure 2B, the “3x, 64–256” represents three identical residual blocks where the sizes of the input and output feature maps are 64 and 256, respectively. The input of the ResNet is the lens region partitioned by Faster R-CNN or Hough transform. The output of the ResNet is the category of normal or infantile cataracts in screening and the severity of infantile cataracts in three-degree grading. Two LPSs can perform lens partition based on slit-lamp images of any size. And the size of the partitioned lens region is larger than the minimum size (256 × 256 pixels) required by the ResNet. Therefore, there is no special requirement on the minimum size and pixels of the slit-lamp images in this study. For a fair comparison, the same 50-layer ResNet and its training parameters are used in all experiments.

### Visualization Heatmaps and t-Distributed Stochastic Neighbor Embedding

To verify the reasonability of diagnosis models, the gradient-weighted class activation mapping (Grad-CAM) visualization technique was employed to generate the heatmaps for highlighting the disease-related regions on which the diagnosis model focused most. The Grad-CAM is an explainable technique for CNN-based models, which utilized the gradients of any target concept flowing into the last convolutional layer to produce a localization map highlighting remarkable regions in the image for predicting the concept (39). The t-distributed stochastic neighbor embedding (t-SNE) (40) was used to present the discrimination ability of high-level features learned by deep learning. The t-SNE is a dimensionality reduction technique that visualizes high-dimensional data by giving each datapoint a location in a two-dimensional map. The separability of different types of datapoints in a two-dimensional map represents the discrimination ability of high-level features in deep learning.

### Evaluation Metrics and Statistical Analysis

This study was conducted using the PyTorch deep learning framework (41), and all models were trained in parallel with four NVIDIA TITAN RTX 24G GPUs. Five-fold cross-validation (30) was applied to calculate the mean and standard deviation of metrics, including accuracy, specificity, sensitivity, F1-Measure, intersection over union (IoU), average precision (AP), receiver operating characteristic (ROC) curve, and area under the ROC curve (AUC), to evaluate the performance of different methods for infantile cataracts detection. The accuracy represents the proportion of correctly classified or graded samples in all samples; the sensitivity and specificity are used to evaluate the probability of misdiagnosis of patients and normal samples, respectively. The F1-Measure, ROC curve, and AUC were used to measure the overall performance of the diagnosis models. The IoU and AP metrics were employed to evaluate the performance of the LPSs, where the IoU represents the ratio between the intersection and union of the prediction and ground truth bounding boxes, and the AP refers to the AP of the RoIs. All statistical analyses were conducted using Python 3.7.8 and the packages of Scikit-learn. The 95% confidence intervals (CIs) for accuracy, specificity, sensitivity, and AUC were calculated with the Wilson score approach.

## Results

### Qualitative Analysis of LPSs

**Figure 3:**
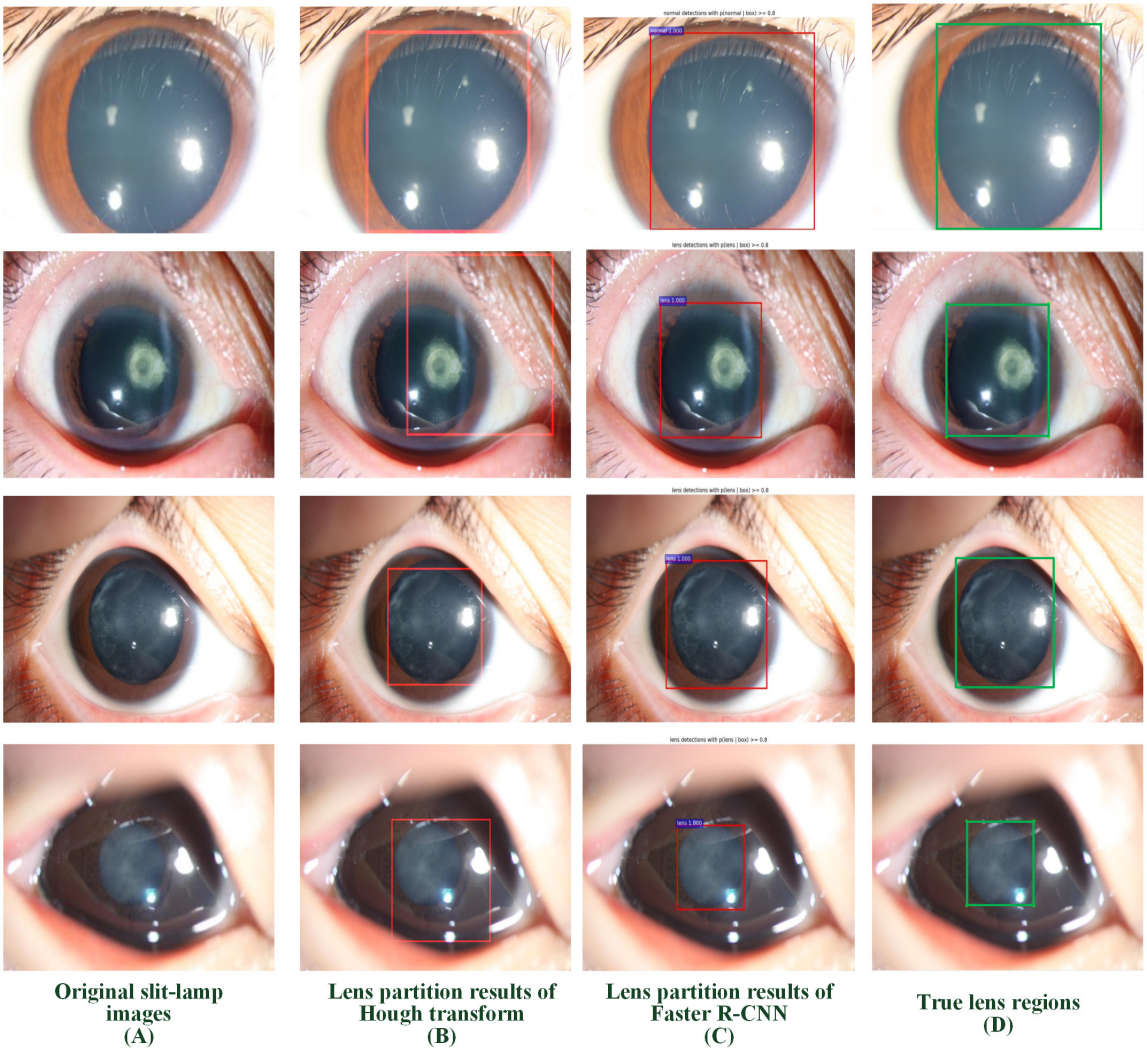
Examples of lens partition using two LPSs. **(A)** Four representative original slit-lamp images. **(B,C)** The automatic lens partition results of Hough transform and Faster R-CNN, where the bold red rectangles denote the boundaries of the partitioned lens. **(D)** The true lens area is manually marked by senior ophthalmologists. LPSs, lens partition strategies.

Two LPSs (Hough transform and Faster R-CNN) were employed to localize the lens RoI. Four representative slit-lamp images, including one normal image and three positive images with various infantile cataracts, were presented to intuitively illustrate the effectiveness of LPSs (Figure 3). Figure 3A denotes the original slit-lamp images, and the bold green rectangles in Figure 3D are the true lens regions manually marked by senior ophthalmologists. Figures 3B,C are the partitioned results of Hough transform and Faster R-CNN, respectively, and the bold red rectangles indicated the lens regions partitioned by the above two methods.

### Quantitative Analysis of LPSs

**Figure 4:**
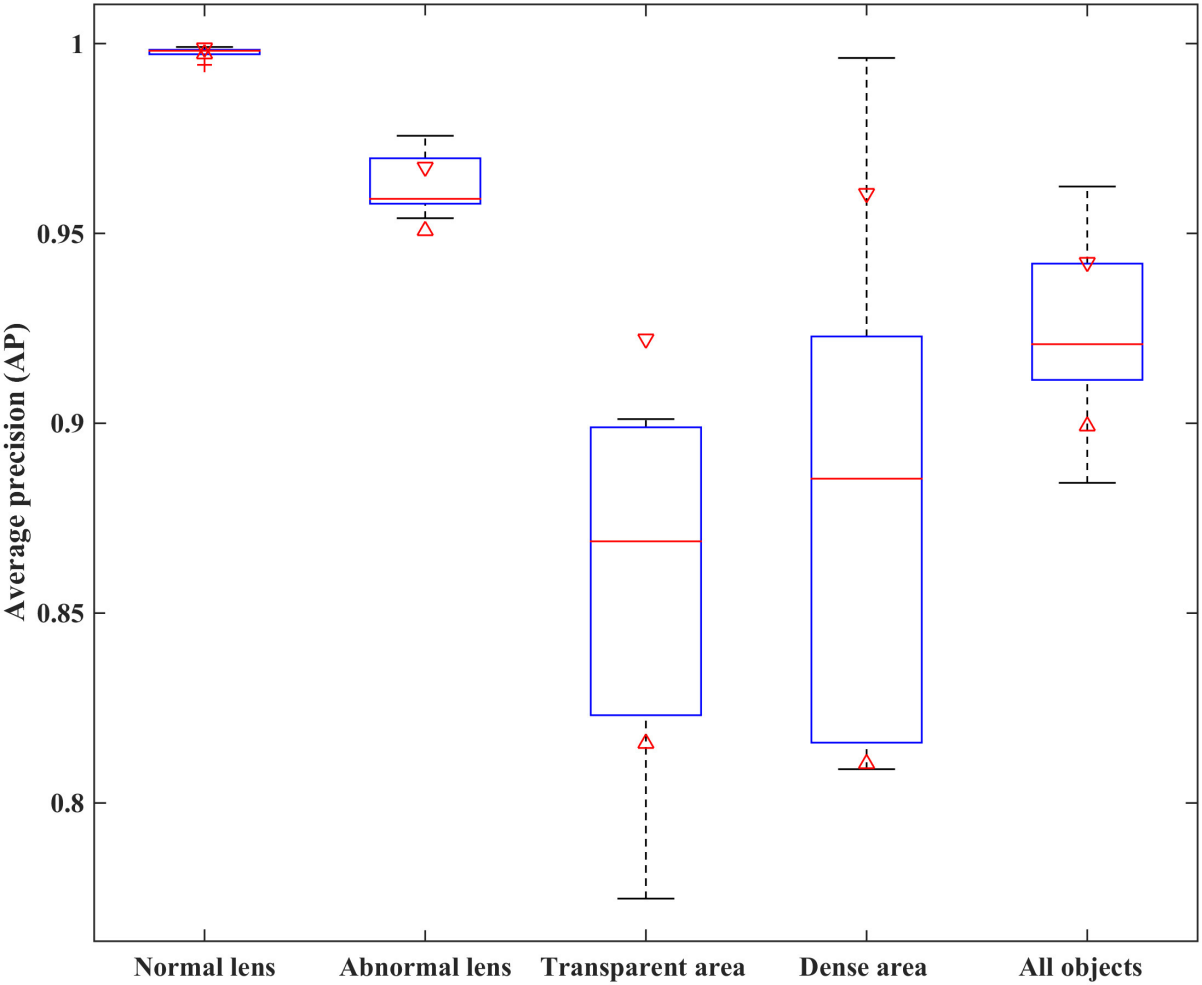
The AP performance comparison of different object regions with the Faster R-CNN partition strategy. AP, average precision.

Two metrics of IoU and AP were employed to quantitatively evaluate the performance of two partition strategies. Using 5-fold cross-validation, we obtained the IoU of normal and abnormal lenses shown in Table 2. For the normal lens, the Hough transform had a mean IoU of 0.897, and the Faster R-CNN had a mean IoU of 0.9419. For the abnormal lens, the Hough transform only obtained a mean IoU of 0.8138, whereas the Faster R-CNN obtained a mean IoU of 0.9107. The boxplot of the AP metric for the Faster R-CNN is shown in Figure 4. The median AP of the partitioned normal lens was > 0.99, and even the median AP of the abnormal lens also achieved more than 0.95. In addition, the median APs of dense regions and transparent regions were 0.8854 and 0.8689, respectively.

**Table 2:** The IoU performance comparison of two LPSs.

**Methods**	**Normal lens** **(mean ± SD)**	**Abnormal lens** **(mean ± SD)**
Hough transform	0.8970 ± 0.0160	0.8138 ± 0.0327
Faster R-CNN	0.9419 ± 0.0037	0.9107 ± 0.0143

*IoU, intersection over union; SD, standard deviation; LPSs, lens partition strategies*.

To evaluate the efficiency of LPSs, we further calculated the time consumption of training and testing procedures. The training time contains a complete transfer learning procedure, and the testing time refers to the average partition time on all test images. As the Hough transform was based on *a priori* hypothesis that the lens is circular, it performed lens partition without any training operation. Thus, the time consumption of training is zero, as shown in Table 3. The training time of the Faster R-CNN based on the transfer learning was 2,906 s. The testing time of the Hough transform is 34.46 s, whereas the Faster R-CNN only needs 0.25 s. For a comprehensive comparison, we listed the average IoU of normal and abnormal lenses in the last column of Table 3. Compared with the Hough transform, the average IoU of the Faster R-CNN was improved by 0.0684.

**Table 3:** The efficiency comparison of two LPSs.

**Methods**	**Running time of training (s)**	**Running time of testing (s)**	**Average IoU of lens** **(mean ± SD)**
Hough transform	0	34.46	0.8554 ± 0.0501
Faster R-CNN	2,906	0.25	0.9238 ± 0.0170

*IoU, intersection over union; SD, standard deviation, LPSs, lens partition strategies*.

### Exploring the Impact of LPSs on the Diagnosis Models

From the above qualitative and quantitative experiments, our study confirmed that the performance of the Faster R-CNN was superior to that of the Hough transform. To evaluate the ultimate impact of LPSs on the diagnosis models, we trained four classifiers for the infantile cataracts screening and three-degree grading based on the CC-Cruiser dataset. Their performance including accuracy, specificity, and sensitivity is shown in Table 4. The ResNet denotes the diagnosis method without using any LPS. The HT-ResNet represents the diagnosis method using LPS based on Hough transform (1, 3). The Faster-RCNN-ResNet represents the diagnosis method using LPS based on Faster R-CNN. For infantile cataracts screening, the ResNet model achieved a 93.08% specificity and a 91.95% sensitivity, and the HT-ResNet model (specificity and sensitivity, 97.28 and 96.83%) was comparable to the optimal Faster-RCNN-ResNet model (98.74 and 97.04%). For area grading, the performance of three models from poor to excellent was as follows: ResNet (77.33 and 81.92%), HT-ResNet (86.63 and 90.75%), and Faster-RCNN-ResNet (94.72 and 94.04%). For density grading, the ResNet achieved a 79.85% specificity and an 81.81% sensitivity, and the performance of the HT-ResNet (91.05and 93.94%) was inferior to that of the Faster-RCNN-ResNet (92.14 and 95.62%). For location grading, the ResNet achieved a 72.01% specificity and an 83.90% sensitivity; the sensitivity of HT-ResNet (93.08%) is close to that of Faster-RCNN-ResNet (94.55%), whereas the specificity of HT-ResNet (82.70%) was inferior to that of Faster-RCNN-ResNet (90.64%). In addition, the standard deviation of Faster-RCNN-ResNet for infantile cataracts screening and three-degree grading was small.

**Table 4:** Quantitative evaluation of different methods using the CC-Cruiser dataset.

	**Metric**	**ResNet**	**HT-ResNet**	**Faster-RCNN-ResNet**
Screening	ACC (mean ± SD) (%)	92.57 ± 0.57	97.07 ± 0.85	97.96 ± 0.32
	SPE (mean ± SD) (%)	93.08 ± 3.74	97.28 ± 1.06	98.74 ± 0.47
	SEN (mean ± SD) (%)	91.95 ± 3.68	96.83 ± 1.83	97.04 ± 0.68
Area grading	ACC (mean ± SD) (%)	79.95 ± 3.06	89.02 ± 0.99	94.33 ± 1.11
	SPE (mean ± SD) (%)	77.33 ± 10.6	86.63 ± 5.81	94.72 ± 2.48
	SEN (mean ± SD) (%)	81.92 ± 7.90	90.75 ± 4.28	94.04 ± 1.78
Density grading	ACC (mean ± SD) (%)	80.95 ± 3.78	92.68 ± 0.61	94.10 ± 1.79
	SPE (mean ± SD) (%)	79.85 ± 6.75	91.05 ± 1.92	92.14 ± 2.30
	SEN (mean ± SD) (%)	81.81 ± 5.24	93.94 ± 1.71	95.62 ± 1.52
Location grading	ACC (mean ± SD) (%)	79.62 ± 4.13	89.28 ± 3.23	93.11 ± 1.06
	SPE (mean ± SD) (%)	72.01 ± 9.31	82.70 ± 6.16	90.64 ± 3.60
	SEN (mean ± SD) (%)	83.90 ± 6.55	93.08 ± 3.87	94.55 ± 1.65

*ACC, accuracy; SPE, specificity; SEN, sensitivity; SD, standard deviation; ResNet, the diagnosis model with the residual convolutional neural network; HT-ResNet, the ResNet diagnosis model with LPS based on Hough transform; Faster-RCNN-ResNet, the ResNet diagnosis model with LPS based on Faster R-CNN*.

### Exploring the Impact of the Sample Size of Multicenter Slit-Lamp Images on the Generalizability of Diagnosis Models

Based on the above comparative experiments, we have obtained the best diagnosis model Faster R-CNN-ResNet. Furthermore, we explored the impact of the sample size of multicenter slit-lamp images on the generalizability of the Faster R-CNN-ResNet. Specifically, we collected the multicenter dataset from other four ophthalmic clinics, employed the Faster R-CNN to crop the lens region, and randomly divided these partitioned images into five equal size subsets. Then, by sequentially adding one to four subsets to the CC-Cruiser dataset, we trained four classifiers and compared their performance on the remaining multicenter testing dataset. Including the original model without any multicenter training dataset, we have obtained five comparison results in Table 5. When only the CC-Cruiser dataset (zero subsets in Table 5) was involved in training, its generalizability on the multicenter testing dataset was not ideal. The performance of the zero-subset model are as follows: infantile cataracts screening [specificity and sensitivity, 92.54% (95% CI, 0.862–0.988) and 96.43% (95% CI, 0.925–1.000)], area grading [78.12% (95% CI, 0.638–0.925) and 88.46% (95% CI, 0.800–0.984)], density grading [81.48% (95% CI, 0.668–0.961), and 87.50% (95% CI, 0.788–0.962)], and location grading [81.82% (95% CI, 0.687–0.950) and 86.00% (95% CI, 0.764–0.956)]. With the addition of multicenter slit-lamp images, the performance of the models was gradually enhanced. The optimal performance (three subsets or four subsets in Table 5) was as follows: infantile cataracts screening [97.01% (95% CI, 0.929–1.000) and 97.62% (95% CI, 0.944–1.000)], area grading [93.75% (95% CI, 0.854–1.000) and 92.31% (95% CI, 0.851–0.996)], density grading [92.59% (95% CI, 0.827–1.000) and 92.86% (95% CI, 0.861–0.996)], and location grading [90.91% (95% CI, 0.811–1.000) and 92.00% (95% CI, 0.845–0.995)]. In addition, the comparison results of the other two methods (ResNet and HT-ResNet) were also obtained after adding four subsets of multicenter slit-lamp images to the training procedure (Table 6). It is not difficult to conclude that the generalizability of the Faster R-CNN-ResNet is superior to the other two methods.

**Table 5:** Performance comparison of Faster-RCNN-ResNet model with different sizes of the multicenter dataset in the training procedure.

	**Metric**	**0-Subset**	**1-Subset**	**2-Subsets**	**3-Subsets**	**4-Subsets**
Screening	ACC (%) (95% CI)	94.70 (0.911–0.983)	96.03 (0.929–0.991)	97.35 (0.948–0.999)	97.35 (0.948–0.999)	97.35 (0.948–0.999)
	SPE (%) (95% CI)	92.54 (0.862–0.988)	95.52 (0.906–1.000)	97.01 (0.929–1.000)	97.01 (0.929–1.000)	97.01 (0.929–1.000)
	SEN (%) (95% CI)	96.43 (0.925–1.000)	96.43 (0.925–1.000)	97.62 (0.944–1.000)	97.62 (0.944–1.000)	97.62 (0.944–1.000)
Area grading	ACC (%) (95% CI)	84.52 (0.768–0.923)	86.90 (0.797–0.941)	91.67 (0.858–0.976)	92.86 (0.874– 0.984)	92.86 (0.874– 0.984)
	SPE (%) (95% CI)	78.12 (0.638–0.925)	81.25 (0.677–0.948)	90.62 (0.805–1.000)	93.75 (0.854–1.000)	93.75 (0.854–1.000)
	SEN (%) (95% CI)	88.46 (0.800–0.972)	90.38 (0.824–0.984)	92.31 (0.851–0.996)	92.31 (0.851–0.996)	92.31 (0.851–0.996)
Density grading	ACC (%) (95% CI)	85.54 (0.780–0.931)	87.95 (0.810–0.950)	90.36 (0.840–0.967)	92.77 (0.872–0.983)	92.77 (0.872–0.983)
	SPE (%) (95% CI)	81.48 (0.668–0.961)	85.19 (0.718–0.986)	88.89 (0.770–1.000)	92.59 (0.827–1.000)	92.59 (0.827–1.000)
	SEN (%) (95% CI)	87.50 (0.788–0.962)	89.29 (0.812–0.974)	91.07 (0.836–0.985)	92.86 (0.861–0.996)	92.8 6 (0.861–0.996)
Location grading	ACC (%) (95% CI)	84.34 (0.765–0.922)	86.75 (0.795–0.941)	91.57 (0.856–0.975)	91.57 (0.856–0.975)	91.57 (0.856–0.975)
	SPE (%) (95% CI)	81.82 (0.687–0.950)	84.85 (0.726–0.971)	90.91 (0.811–1.000)	90.91 (0.811–1.000)	90.91 (0.811–1.000)
	SEN (%) (95% CI)	86.00 (0.764–0.956)	88.00 (0.790–0.970)	92.00 (0.845–0.995)	92.00 (0.845–0.995)	92.00 (0.845–0.995)

*ACC, accuracy; SPE, specificity; SEN, sensitivity; CI, confidence interval; 4-subsets, the diagnosis model with four subsets multicenter slit-lamp images included in the training procedure*.

**Table 6:** Evaluation of generalization of different methods on the multicenter dataset.

	**Metric**	**ResNet**	**HT-ResNet**	**Faster-RCNN-ResNet**
Screening	ACC (%) (95% CI)	90.73 (0.861–0.954)	93.38 (0.894–0.973)	97.35 (0.948–0.999)
	SPE (%) (95% CI)	91.04 (0.842–0.979)	94.03 (0.884–0.997)	97.01 (0.929–1.000)
	SEN (%) (95% CI)	90.48 (0.842–0.968)	92.86 (0.873–0.984)	97.62 (0.944–1.000)
Area grading	ACC (%) (95% CI)	77.38 (0.684–0.863)	88.10 (0.812–0.950)	92.86 (0.874– 0.984)
	SPE (%) (95% CI)	75.00 (0.600–0.900)	87.50 (0.760–0.990)	93.75 (0.854–1.000)
	SEN (%) (95% CI)	78.85 (0.677–0.899)	88.46 (0.798–0.971)	92.31 (0.851–0.996)
Density grading	ACC (%) (95% CI)	75.90 (0.667–0.851)	87.95 (0.810–0.950)	92.77 (0.872–0.983)
	SPE (%) (95% CI)	74.07 (0.576–0.906)	88.89 (0.770–1.000)	92.59 (0.827–1.000)
	SEN (%) (95% CI)	76.79 (0.657–0.878)	87.50 (0.788–0.962)	92.86 (0.861–0.996)
Location grading	ACC (%) (95% CI)	74.70 (0.653–0.841)	83.13 (0.751–0.912)	91.57 (0.856–0.975)
	SPE (%) (95% CI)	69.70 (0.540–0.854)	78.79 (0.648–0.927)	90.91 (0.811–1.000)
	SEN (%) (95% CI)	78.43 (0.665–0.895)	87.13 (0.764–0.956)	92.00 (0.845–0.995)

*ACC, accuracy; SPE, specificity; SEN, sensitivity; CI, confidence interval; ResNet, the diagnosis model with the residual convolutional neural network; HT-ResNet, the ResNet diagnosis model with LPS based on Hough transform; Faster-RCNN-ResNet, the ResNet diagnosis model with LPS based on Faster R-CNN*.

**Figure 5:**
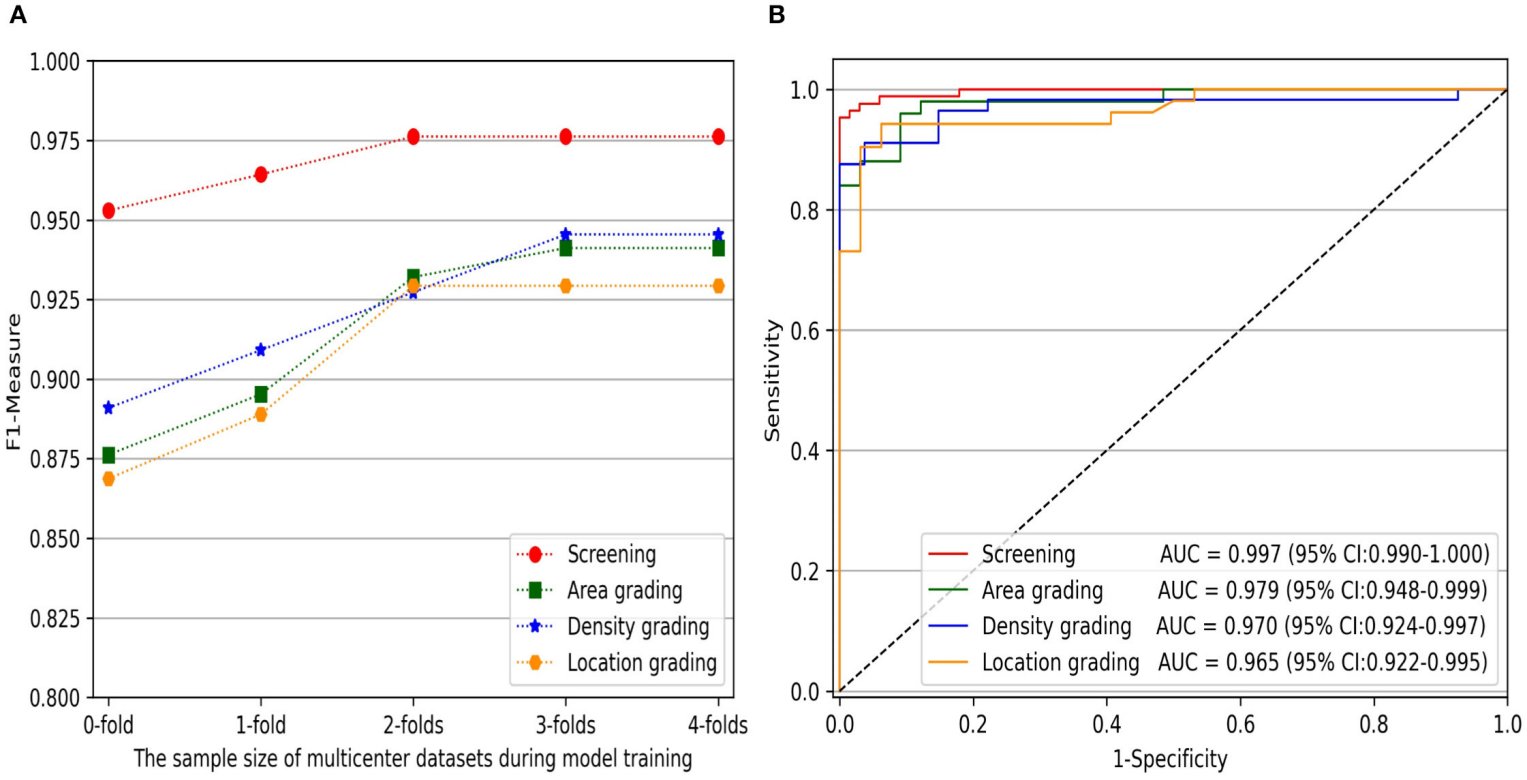
The impact of the sample size of multicenter training datasets on infantile cataracts screening and three-degree grading. **(A)** The F1-Measure curves with the increase of multicenter training samples for screening and three-degree grading. **(B)** The ROC curves for screening and three-degree grading. ROC, receiver operating characteristic curve; AUC, area under the ROC curve.

Furthermore, to comprehensively evaluate the impact of the sample size of multicenter slit-lamp images on the generalizability of models, we presented the F1-Measure curve with the addition of the multicenter training dataset shown in Figure 5A. From zero to two subsets, the performance of screening and three-degree grading increased rapidly. From two to four subsets, the screening and the location grading were stable, whereas the area and density grading were also slightly improved. Furthermore, we showed the ROC curves of infantile cataracts screening and three-degree grading when three subsets multicenter slit-lamp images were included in the training procedure. As shown in Figure 5B, the ROC of the screening was closer to the upper-left corner with an AUC of 0.997 (95% CI, 0.990–1.000). Among the three-degree grading, the area grading showed the optimal result with an AUC of 0.979 (95% CI, 0.948–0.999), followed by the density grading with an AUC of 0.970 (95% CI, 0.924–0.997), and location grading with an AUC of 0.965 (95% CI, 0.922–0.995). In addition, we obtained a 0.9246 AP and a 0.9600 IoU for lens partition on multicenter slit-lamp images, which further verified the generalizability of the Faster R-CNN in the multicenter dataset.

### Interpretability Analysis of Infantile Cataracts Screening and Three-Degree Grading Models

**Figure 6:**
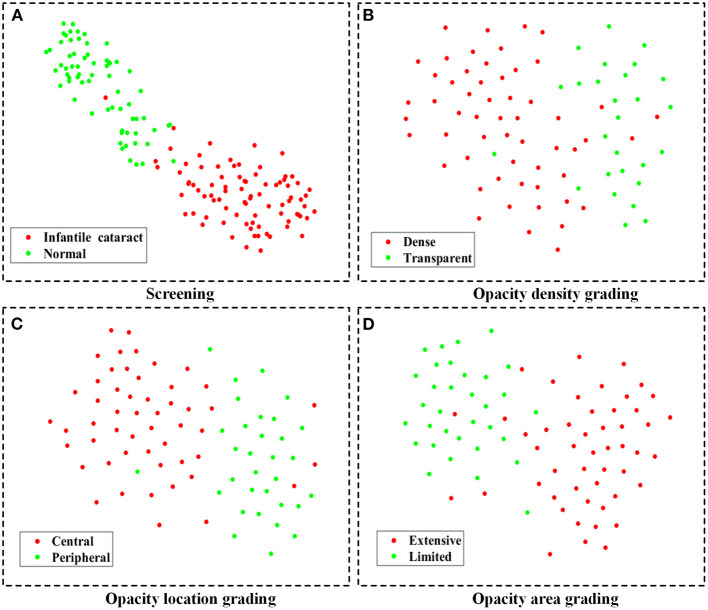
The feature maps of the multicenter testing dataset for screening and three-degree grading using t-SNE. **(A–D)** Two-dimensional feature maps for screening, opacity density grading, opacity location grading, and opacity area grading, respectively. The red dots represent infantile cataract patients in screening or severe patients in grading, and the green dots denote normal samples or mild infantile cataract patients. t-SNE, t-distributed stochastic neighbor embedding.

**Figure 7:**
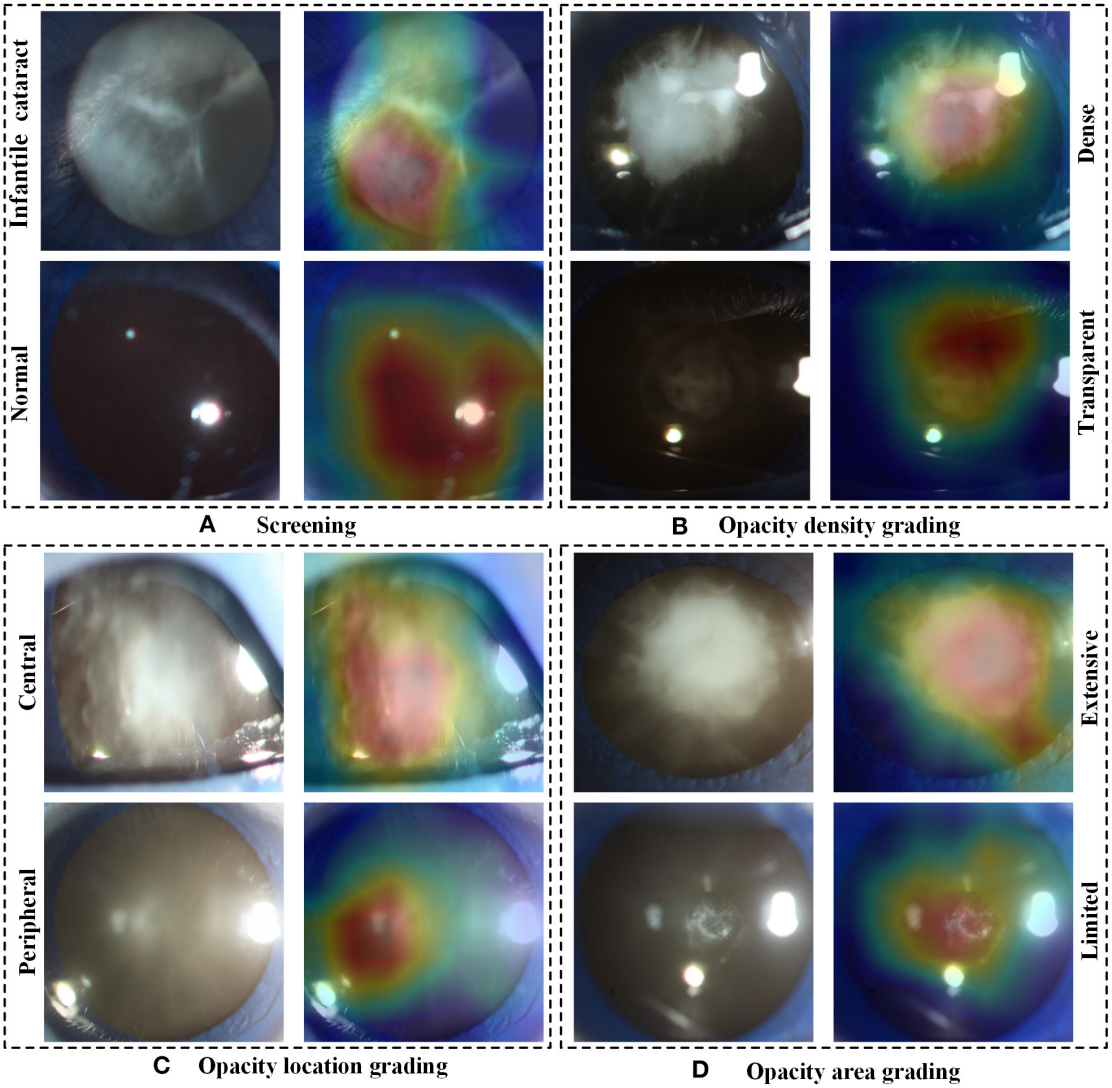
The representative heatmaps of infantile cataracts screening and three-degree grading using Grad-CAM. **(A–D)** The representative positive and negative slit-lamp images and their corresponding visualization heatmaps for infantile cataracts screening, opacity density grading, opacity location grading, and opacity area grading, respectively. In each figure, the upper row indicates infantile cataract patients in screening or severe patients in grading, and the lower row represents normal images or mild infantile cataract patients. Grad-CAM, gradient-weighted class activation mapping.

In the multicenter testing dataset, the high-level features extracted from the screening and three-degree grading models, including 67 normal samples and 84 infantile cataract samples with various severities, were mapped into a two-dimensional space to visually demonstrate their discrimination (Figure 6). Visualized maps of the screening (Figure 6A), opacity density (Figure 6B), opacity location (Figure 6C), and opacity area (Figure 6D) are presented separately in Figure 6, where the red dots represent infantile cataract patients in screening or severe patients in grading, and the green dots denote normal samples or mild infantile cataract patients. Furthermore, eight representative slit-lamp images and their visual heatmaps are displayed in Figure 7. For screening, the upper and lower rows are infantile cataract patients and normal samples (Figure 7A), both of which include the original slit-lamp image and the corresponding heatmap. Similarly, in each subheatmap of opacity density (Figure 7B), location (Figure 7C), and area (Figure 7D), the upper row represents severe infantile cataract patient, and the lower row denotes mild patient.

## Discussion

In this study, we proposed an effective LPS based on deep learning Faster R-CNN for improving the generalizability of the infantile cataracts screening and three-degree grading. The impacts of LPSs and multicenter slit-lamp images on the performance of the diagnosis models were investigated. Qualitative and quantitative experiments demonstrated that the Faster R-CNN was the optimal LPS with higher accuracy and efficiency compared to the Hough transform. The sample size of the multicenter slit-lamp images had a significant impact on the performance of diagnosis models. With the addition of multicenter slit-lamp images, the performance of the diagnosis models was gradually enhanced. Through comparative experiments, the minimal training sample size of multicenter images was achieved to ensure the better generalizability of diagnosis models. Moreover, the Grad-CAM and t-SNE techniques provided an interpretable path for the diagnosis of infantile cataracts, further validating the effectiveness of the Faster R-CNN.

The performance of the Faster R-CNN was superior to that of the Hough transform on both normal and abnormal lenses. First, the qualitative experiment presented that the lens partitioned by Faster R-CNN perfectly matched the true lens marked by senior ophthalmologists, and the difference between them is small. Previously, our previous studies (1, 3) reported the lens partition method HT-ResNet based on Hough transform. However, there was a large deviation between the lens regions partitioned by Hough transform and the true lens regions, especially on the abnormal lenses from rows 2 and 4 of Figure 3. Second, the average IoU of lens partition based on the Faster R-CNN was higher when compared to the Hough transform. Especially on the abnormal lens, the average IoU of the Faster R-CNN was improved by 0.0969. Third, regardless of the normal or abnormal lenses, the distribution of the AP boxplot of the Faster R-CNN was compact, and their median exceeded 95%. The above analysis indicated that the lens partition of the Faster R-CNN was effective. It removed most of the noises around the lens, which was conducive to improving the performance of the diagnosis models. Furthermore, according to the grading standard of opacity density, senior ophthalmologists also marked the dense and transparent lesion regions, and then we calculated the AP of these regions in the same way. However, the APs of the dense and transparent lesion regions showed lower and more scattered characteristics in the boxplot. We speculated that this result was due to the scattered distribution of opacity lesion regions of infantile cataracts.

The lens partition efficiency of Faster R-CNN is high. Although the training time of the Hough transform is zero, its average testing time exceeded half a minute. In contrast, the average testing time of the Faster R-CNN was only 0.25 s. As the training procedure can be performed on the local GPU server in advance, the testing time will not be affected after the trained model is deployed in ophthalmic clinics. Therefore, the Faster R-CNN can be applied to real-time lens partition for infantile cataracts.

The LPS directly affected the performance of the diagnosis models. First, for infantile cataracts screening, the accuracy, specificity, and sensitivity of the two LPSs have been improved approximately by 4–5%, which were slightly better than the ResNet without using any LPS. Second, for infantile cataracts three-degree grading, compared with the ResNet, the performance of the Faster-RCNN-ResNet was greatly improved, followed by HT-ResNet. For example, the accuracy, specificity, and sensitivity of the Faster-RCNN-ResNet were increased by 14.38, 17.39, and 12.12% in the opacity area grading, respectively, and those indicators of the HT-ResNet were increased by ~9%. Similar conclusions were presented on the other grading of opacity density and location. Third, in all grading, the Faster-RCNN-ResNet was superior to the HT-ResNet. It was worth mentioning that the specificity of the Faster-RCNN-ResNet was improved by 8.09 and 7.94% in the opacity area and location, respectively, when compared to the HT-ResNet method.

The multicenter slit-lamp images affected the generalizability of diagnosis models because fewer single-center images cannot represent the diversity of real data in multicenter clinics. In our previous study, we had verified that the model trained with only the CC-Cruiser dataset did not perform well in other clinical institutions (28). In this study, with the addition of multicenter slit-lamp images to the CC-Cruiser dataset, although the performance improvement of infantile cataracts screening was subtle, the performance of three-degree grading was greatly improved. As shown in Figure 5, when more than two subsets of the multicenter dataset were added, the F1-Measure indicator of three-degree grading tended to be stable. This result is attributed to the following three factors. First, the difference was large between abnormal images but small between normal images. Three screening models distinguished the normal lens from the abnormal lens with high accuracy. Second, if there is no or only a small size of multicenter data involved in training, the characteristics of multicenter slit-lamp images cannot be adequately learned by models. In addition, the noises and heterogeneity between abnormal images collected in multicenter were large. These factors affected the generalizability of models in three-degree grading.

Although deep learning, like a black box, was applied in extensive disease diagnosis, their insufficient interpretability characteristic was criticized by doctors and patients. Exploring the reasons for the rationale of deep learning models can facilitate their acceptance and application. In this study, we explored the discrimination ability of high-level features extracted from deep learning using the t-SNE technique (Figure 6). The features of the infantile cataracts screening mapped to a two-dimensional space were highly separable, whereas the separability of the three-degree grading was slightly weak. These results were consistent with our quantitative testing results of infantile cataracts screening and grading. Furthermore, we performed heatmap visualization on the multicenter testing dataset (67 normal and 84 abnormal slit-lamp images) using the Grad-CAM technique. Eight representative images were presented to illustrate the regions contributing the most to the outcome of the models. Inspiringly, the visualization results showed that all abnormal images highlighted the lesion regions of infantile cataracts, and normal images highlighted the lens region, which further corroborated the reasonability of the proposed diagnosis system. Interpretability analysis provided strong evidence for the acceptance of the Faster R-CNN-ResNet in ophthalmic clinics.

Several limitations exist in this study. First, although both normal and abnormal lenses were partitioned, the specific lesions regions were not accurately partitioned because the distribution of the lesions was diffuse and sparse. Segmentation methods may be another path for addressing lesions regions. The dense labeling and segmentation methods of lesions need to be further studied, which may provide a more accurate diagnosis for infantile cataracts. Second, only slit-lamp images had been studied for the automatic diagnosis of infantile cataracts, the diagnostic system may miss a few patients whose lesions occur on the back of the lens. It is necessary to explore multimodality image fusion methods to comprehensively evaluate the severity of infantile cataracts. Third, this study mainly focused on the severities of infantile cataracts from three-degree grading: opacity area, density, and location. However, there are other types of infantile cataracts from other grading perspectives, such as punctate cataract, lamellar cataract, posterior polar cataract, and so on. The performance of LPSs on different types of infantile cataracts will be explored in future research. In addition, our AI diagnosis system did not fully consider infantile cataracts with wobbly movements, which will be further explored by video detection methods.

## Conclusion

This study presented a feasible LPS and diagnosis system for infantile cataracts that can be applied to the multicenter slit-lamp images. An appropriate LPS and sample size of multicenter images can help to improve the reliability and generalizability of the diagnostic system. Qualitative and quantitative experiments verified that the Faster R-CNN-ResNet was superior to other conventional methods in infantile cataracts screening and three-degree grading. Furthermore, the discrimination of high-level features and the most relevant heatmaps were visualized using t-SNE and Grad-Cam techniques, respectively, which made the diagnosis results by the model interpretable. This study also provided a valuable reference for the analysis of other rare diseases and improved the generalizability of deep learning technology in clinical applications.

## Data Availability Statement

The raw data supporting the conclusions of this article will be made available by the authors, without undue reservation.

## Ethics Statement

The studies involving human participants were reviewed and approved by the Institutional Review Board of Zhongshan Ophthalmic Center of Sun Yat-sen University (Guangzhou, Guangdong, China). Written informed consent to participate in this study was provided by the participants' legal guardian/next of kin.

## Author Contributions

JJ, SL, MZ, RL, and HL were responsible for the initial plan and study design. RL, ZLi, and JC collected the data. JC, RL, DL, and ZLin labeled the data. JJ, SL, MZ, XW, and JY performed the experiments and analyzed the data. JJ wrote the paper. JJ, ZLi, JG, and HL revised it. All authors read and approved the final manuscript.

## Conflict of Interest

The authors declare that the research was conducted in the absence of any commercial or financial relationships that could be construed as a potential conflict of interest. The handling Editor declared a past collaboration with one of the authors HL.
